# Miscellaneous Items and News

**Published:** 1857-08

**Authors:** 


### Miscellaneous Items and News.

A lady in Richmond, Va., died in about twenty-four hours from a spider's bite upon the cheek.——Sixty-five is the age at which the authorities of one of our Medical Colleges pronounce a medical teacher superannuated.——A blind man in Paris having been restored to sight by a renowned oculist, out of charity, sued the operator for 20,000f. damages, for destroying his profession as blind beggar.——*Glycerine*

preserves animal and vegetable substances from putrefaction or decomposition.——Jabez Hogg, M. R. C. S., has issued a work upon the microscope, which is very little else than a wholesale plagiarism from "The Microscopist," by one of our own countrymen, Dr. Joseph H. Wythes. This is emphatically "going the *whole* hog;" but as Jabes spells his name with a double g, we suppose he considers himself a *double hog*, and hence has not transcended his swinish prerogatives. ——Sir Marshall Hall has in a gentlemanly manner yielded the credit of the discovery and designation of the *excito-secretory system of spinal nerves* to Dr. Henry F. Campbell, of Augusta, Georgia. Dr. Hall can well afford to be just, since he needs nothing more than he already possesses in the way of deserved reputation; but it is so common a thing for men really great in many regards to exhibit little meannesses in such matters that we are ever glad when one proves himself above them.——The Medical College of Ohio and the Miami Medical College have finally been consolidated under the name and style of the former. Drs. Judkins, Mendenhall, Foote, und Comegys, of the Miami school, go into the new Faculty, while Drs. Armor, Tate, Warden and Marshall, of the Ohio school, retire.——A lady in Otsego county, N. Y., presented her husband four living chileren at one birth.——The London Homœopathic Hospital has finally been abandoned as a failure. Ditto in Chicago.——A patient of Dr. A. S. McGregor, of Gasconade, Mo., gave birth August 10, 1856, to a stillborn child, twenty-one days after to a living child, and twenty-one days thereafter to still another.——"Her Majesty, Queen Victoria, has been graciously pleased to bestow the honors of knighthood on her chief accoucher, Dr. Locock. Punch suggests, however, that this distinguished gentleman deserved after his arduous duties to have been elevated to the peerage, under the title of *Lord Deliver us*."

